# Effect of Ozone Treatment on Deoxynivalenol and Wheat Quality

**DOI:** 10.1371/journal.pone.0147613

**Published:** 2016-01-26

**Authors:** Li Wang, Huili Shao, Xiaohu Luo, Ren Wang, Yongfu Li, Yanan Li, Yingpeng Luo, Zhengxing Chen

**Affiliations:** 1 Key Laboratory of Carbohydrate Chemistry and Biotechnology Ministry of Education, State Key Laboratory of Food Science and Technology, Collaborative Innovation Center for Food safety and quality control, Jiangnan University, Wuxi, 214122, Jiangsu province, China; 2 National Engineering Laboratory for Cereal Fermentation Technology, School of Food Science and Technology, Jiangnan University, Wuxi, 214122, Jiangsu province, China; Murdoch University, AUSTRALIA

## Abstract

Deoxynivalenol (DON) is a secondary metabolite produced by *Fusarium* fungi, which is found in a wide range of agricultural products, especially in wheat, barley, oat and corn. In this study, the distribution of DON in the wheat kernel and the effect of exposure time to ozone on DON detoxification were investigated. A high concentration of toxin was found in the outer part of the kernel, and DON was injected from the outside to the inside. The degradation rates of DON were 26.40%, 39.16%, and 53.48% after the samples were exposed to 75 mg/L ozone for 30, 60, and 90 min, respectively. The effect of ozonation on wheat flour quality and nutrition was also evaluated. No significant differences (*P* > 0.05) were found in protein content, fatty acid value, amino acid content, starch content, carbonyl and carboxyl content, and swelling power of ozone-treated samples. Moreover, the ozone-treated samples exhibited higher tenacity and whiteness, as well as lower extensibility and yellowness. This finding indicated that ozone treatment can simultaneously reduce DON levels and improve flour quality.

## Introduction

Deoxynivalenol (DON), a secondary metabolite produced by *Fusarium* fungi, is a common contaminant in wheat, barley, oat, and corn[1,2]. *Fusarium* head blight, a fungal disease caused by several *Fusarium* species, occurs during wheat plant growth under humidity conditions suitable for fungal growth and temperate weather[3]. This disease causes two forms of agricultural damage: reduction in harvest because of grain shriveling and threat to food safety because of DON accumulation in grains by several *Fusarium* species[4]. Human and animal exposure to DON via ingestion of contaminated food can induce acute and chronic effects, such as immunosuppression, neurotoxicity, embryotoxicity, and teratogenicity[5–7]. The International Agency for Research on Cancer categorized DON as a Group 3 carcinogen[8]. Thus, safe, economical, and cost-effective technologies must be developed to reduce fungal growth and remove DON in wheat grains while mitigating post-harvest losses. DON detoxification involves physical, chemical, and biological methods[9–12]. However, each technique exhibits certain limitations, such as nutrition loss, sensory attribute reduction, inconvenient operation, and high equipment cost, thereby hindering their practical applications.

Ozone has been widely used in the food industry as an effective antimicrobial agent for DON detoxification because of its potential oxidizing capacity[13]. The use of ozone in gaseous or aqueous phase as an antimicrobial agent has been approved by the US FDA according to the Federal Register provisions[14] and good manufacturing practices for processing and storage of food, bottled water, apple juice, and cider[15]. The advantages of ozone over traditional fumigants are as follows: (a) ozone rapidly decomposes (half-life of 20–50 min) to molecular oxygen, (b) leaves no residue, (c) can be generated on site, and (d) requires no storage and subsequent disposal of chemical containers[16]. Ozone can also kill pests in food and inactivate microbes, including bacteria, fungi, and viruses[17–19]. As such, ozone has been widely used to control the growth of various fungi in laboratory-scale trials in food, such as red pepper, wheat, dried fig, and peanut[20–24]. Since 1960, numerous studies have suggested that ozone treatment effectively degrades AFB_1_, the most toxic compound among various fungi; moreover, the degradation products of AFB_1_ after reacting with ozone has been widely studied[25–28]. However, data on DON degradation after ozone treatment are limited, and few studies have examined the effect of ozone treatment on the processing performance of wheat flour[29,30]. The limited studies on this field could be due to legislation that prohibits chemical treatment and uncertainty over the identity of reaction products.

Ozone treatment, a chemical detoxification method, can effectively control fungi in grains and may affect the quality of wheat in several aspects. Ozone can induce oxidation and/or modification of the physicochemical constituents of grains through lipid and starch oxidation, protein modification, grain discoloration, or changes in flour functionality. Sandhu et al.[31] found that ozone treatment can oxidize lipids, enhance brightness, reduce yellow hue, and increase peak and setback viscosities of flour. Violleau et al.[32] reported that ozonation can efficiently modify the technological properties of wheat grain.

Wheat and wheat products are consumed as food by approximately half of the world’s population[15]; as such, the property of wheat after ozone treatment must be elucidated for human dietary purposes. A commercially acceptable method for detoxifying DON must be developed for application in the food industry. The present study aimed to (1) investigate the distribution of DON in the wheat kernel and the effects of duration of ozone exposure on DON detoxification to propose a practical method for ozonation detoxification, and (2) assess wheat flour quality and nutrition changes caused by ozonation.

## Materials and Methods

### Materials

#### Wheat samples

Wheat samples naturally contaminated with DON (*Wanmai 50*) were obtained from Suzhou City, Anhui Province, People’s Republic of China (E116°09′–118°10′, N 33°18′ –34°38′) at the end of June 2013. The DON level reached 1.69 mg/kg.

### Methods

#### Ozone treatment of wheat grains

Grains (20 kg) were subjected to ozone treatment using the procedure of Luo et al.[28] with minor modification. Wheat samples were placed in an airtight stainless steel reactor (70 cm in diameter and 140 cm in height). Ozone was produced using YG100 ozone generator (Beijing Shanmeishuimei Industry Co. Ltd., Beijing, China). Ozone gas (75 mg/L) was introduced into the ozone detector (Ideal-2000, Ideal Co. Ltd., Zibo, China) and then into the bottom of the reactor via a glass tube. Another ozone detector was connected to the outlet of the reactor to maintain the concentration of ozone. A disposal apparatus (model DT250, Qingdao Guolin Industry Co. Ltd., Qingdao, China) was used to rapidly convert residual ozone into oxygen after the reaction with the samples. The ozone detector was used to detect ozone concentration in gas flow from the disposal apparatus. Wheat grains were exposed to ozone for 0, 30, 60, or 90 min at 25°C under 75% RH. During the treatment, grains were agitated in the reactor by using a central endless screw. After the ozone treatment, the grains were stored in clean polyethylene bags, which were either sealed and stored at 4°C for further analysis or used in milling.

#### Milling

Water (2% to 5% of dry weight) was added to the treated wheat grains to reach 15% to 16% (w/w) moisture content, depending on the initial water content. Each wheat sample was milled on a Buhler laboratory experimental mill (MLU-202) in accordance with the International Approved Methods 26–21.02[33] to obtain three samples of breaking flours (1B, 2B, and 3B), three samples of middling flours (1M, 2M, and 3M), and two samples of outer-layer fractions (bran and shorts). The milling room was maintained at 23°C–25°C under 40%–50% relative humidity. The milling recovery was 93% to 95%. The homogenous blend of 1B, 1M, 2B, 2M, 3B, and 3M fractions in a plastic bag (70 cm × 100 cm) was shaken 20 times to obtain a patent flour sample.

#### DON analysis

Wheat grain samples were analyzed using immunoaffinity columns for the cleaning step and LC/UV for detection according to the Chinese National Standard [34] with minor modifications. Briefly, 25 g of the sample was placed into a 250 mL Erlenmeyer flask and added with 100 mL of acetonitrile–water (84/16, V/V). The solution was mixed for 30 min under 200 r/min, filtered, and cleaned using a solid-phase extraction cleanup column (Mycosep227^#^, Romer Labs Inc., USA). Eluate (2 mL) was collected and evaporated using a heating block device at 50°C in a gentle nitrogen stream. Dry extract was then redissolved in 1 mL of acetonitrile–water (6:94, V/V) and filtered through a 0.22 μm filter membrane prior to high-performance liquid chromatography (HPLC) analysis.

The extract (20 μL) was injected into the LC/UV system at 218 nm, and the mobile phase was delivered at a constant flow rate of 0.8 mL/min. The total run time was 20 min, and the retention time of DON was 12.3 min. DON level was determined by comparing the measurement of the peak area at the DON retention time and that of the standard solutions used for the calibration curve (0.2, 0.5, 1, 3, 5, 10, 15, and 20 μg/mL; correlation *r* = 0.997).

#### Protein and lipid analyses and alveographic measurements

Protein content of wheat flour was determined according to the AACC International Approved Methods 46–12.01 by using nitrogen with a protein conversion of (N) × 5.7[33]. Alveographic measurements were performed based on Standard NF ISO 5530–4[35] with a Chopin NG alveograph (Chopin Technologies, Paris, France). The alveograph parameters were automatically recorded using the Chopin Alveolink-NG software; these parameters included the maximum overpressure or tenacity (P) needed to blow the dough bubble; abscissa at rupture (L), which measures dough extensibility; index of swelling (G) (measured as the square root of the volume of air necessary to inflate the dough bubble until it ruptures); deformation energy of dough (W), which represents the energy necessary to inflate the dough bubble to the point of rupture; deformation curve (P/L), and elasticity index (Ie). The alveograph W value could be considered as the indicator of gluten strength, whereas the alveograph P/L value represents the balance of elasticity and extensibility[36].

Fatty acid value was evaluated based on the method described by Rose et al.[37] to determine the effect of ozone exposure on fat components of wheat flour. The final results were expressed in terms of milligrams of KOH required to neutralize free fatty acid in 100 g of wheat flour (dry basis).

#### Color measurement of wheat flour

Color of the wheat flour samples was evaluated using Ultra Scan Pro 1166 (Hunterlab, USA) and expressed as L*, a*, and b*. L* denotes a measurement of brightness (0–100), a* represents the red–green coordinates (− is green and + is red), and b* measures the blue–yellow coordinates (− is blue and + indicates yellowness) of the product. All these tests were conducted in triplicate.

#### Analysis of amino acids by HPLC

Amino acids were analyzed using HPLC according to the method developed by Yuetong et al.[38] with minor modifications. A sample (300 mg) was weighed into a screw-capped test tube and added with 6 M hydrochloric acid (8 mL) to determine amino acid content. The tubes were capped and hydrolyzed for 24 h at 110°C. After hydrolysis, the mixture was transferred to a 25 mL volumetric flask with ultrapure water and then decanted through filter paper (Whatman No. 1). The filtrate was evaporated to dryness under vacuum, and the hydrolysates were reconstituted in 1 mL of 0.02 M hydrochloric acid.

Analyses were performed on an Agilent 1100 HPLC (Palo Alto, CA, USA) equipped with a UV detector. Chromatographic separation was performed using an ACE HPLC column (C18-HL; HiChrom, Reading, UK) with a particle size of 5 μm (250 mm × 4.6 mm). Detection wavelength was 338 nm, and the column temperature was maintained at 40°C. The mobile phases used were as follows: (A) 0.10 mol/L sodium acetate buffer (pH 7.2 adjusted with acetic acid solution, 5% v/v) containing 0.0225% (v/v) triethylamine and 0.5% (v/v) tetrahydrofuran; and (B) 40:40:20 (v/v) mixture of acetonitrile, methanol, and 0.36 mol/L sodium acetate (pH 7.2 adjusted with acetic acid solution, 5% v/v). Amino acids were eluted under the following conditions: 8% B for 27.5 min and 60% B for 4 min (1.0 mL/min flow rate); 100% B for 1.5 min (1.5 mL/min flow rate); and 100% B for 2 min and 8% B for 1.5 min (1.0 mL/min flow rate). The target compounds were identified based on the retention times and UV-Vis spectral characteristics of the corresponding derivatized standards. Results were calculated and reported as gram of amino acid per 100 g of the sample.

#### Starch analysis

Starch content of flour was estimated using Megazyme kits (Megazyme International Ireland Ltd., Ireland) according to the approved AACC method 76–13[33]. Starch was isolated from wheat flour samples by using the procedures reported by Rupollo et al.[39] and Vanier et al.[40]. Starch was collected and dried in an oven at 40°C for 12 h. The starch powder was passed through a 100-mesh sieve and stored at ambient temperature.

Carboxyl content of the isolated starch was determined according to the procedure of Chattopadhyay et al.[41]. Carbonyl content of the isolated starch obtained from wheat grain was determined based on the titrimetric method reported by Smith[42]. Swelling power was determined on wheat flour samples by using the method described by McCormick et al.[43]. Swelling power was calculated as sediment weight divided by dried sample weight.

### Statistical analysis

Data were evaluated by analysis of variance in SPSS 20.0 (SPSS Inc., Chicago, IL, USA). F-test was considered significant at *P* ≤ 0.05. Means were separated using Fisher’s protected least significant differences at *P* = 0.05.

## Results and Discussion

### DON analysis after ozone treatment

The distribution of DON in wheat kernel is shown in Table 1. The wheat grain sample, *Wanmai 50*, contained 1.69 mg/kg DON. Most toxins (DON) in the grain was distributed in the 1M flour, followed by bran, as shown in the “Fraction recovery” values presented in Table 1 [fraction recovery = fraction weight (%) × DON concentration (mg/kg)]. For example, the DON value was 0.44 mg/kg in 1M flour and 0.33 mg/kg in bran. The DON concentration gradually increased from 1B flour to 3B flour and then from 1M flour to 3M flour (Table 1). The retention of DON in the patent flour for human consumption was 53.25%. In the milled fractions of wheat, 1B and 1M fractions were located closest to the center of the endosperm, whereas 3B and 3M fractions were closest to the outer part of the kernel. In this study, a high toxin concentration was found in the outer part of the kernel, and DON level was higher inside than that outside the kernel. Most DON remained in the outer layer, where the majority of toxins accumulated. Despite different approaches used in various studies, researchers reported similar trends in mycotoxin distribution in different milled wheat fractions. Young et al. [44] indicated that the highest fungal infection is found on or near the kernel surface. Lancova et al.[45] demonstrated that the DON level of flour is approximately half the concentration of the whole wheat, and DON levels in bran can be two or more times higher than those in the wheat kernels; this finding indicated the presence of high toxin concentrations in the outer part of the kernel. During dry milling, toxins may concentrate on the bran and germ layers[46–48]. Interestingly, the total amount of DON in all fractions decreased by 25.44% compared with that in the whole wheat; this finding is consistent with the result reported by Zhang et al.[49] on the fate of DON during wheat milling. This reduction could not be attributed to experimental error. Moreover, Jackson et al.[50] reported that extrusion of maize-based foods, especially those with added glucose, leads to substantial decreases in AFB_1_; in addition, 23%–38% AFB_1_ in extruded grits with and without added glucose is bound to the component(s) of maize grits. We hypothesize that wheat grains are subjected to extrusion and shear forces of the grinder roll during milling, and the temperature increases with prolonged milling time. Thus, the mycotoxin structure may be modified by the interaction with wheat components.

**Table 1:** Milling properties of wheat and distribution of DON.

	Fraction weight (%)	DON concentration (mg/kg)^A^	Fraction recovery (mg/kg) ^B^
**Wheat kernel**		1.69^a^	
**1B flour**	8.5	0.75^b^	0.06
**2B flour**	5.7	0.81^c^	0.05
**3B flour**	1.7	0.85^c^	0.01
**1M flour**	51.6	0.86^c^	0.44
**2M flour**	7.0	1.13^d^	0.08
**3M flour**	1.2	2.78^e^	0.03
**Shorts**	8.4	2.94^f^	0.25
**Bran**	15.9	2.05^f^	0.33
**Total**	100		1.26
**Patent flour**	75.7	0.90^cd^	0.68
**Recovery after milling (%)**^C^			74.56
**Retention in patent flour (%)**		53.25	

Means followed by the same small letters within columns are not significantly different (*P* > 0.05).

^A^ Mean values (*n* = 3) are shown.

^B^ Calculated by fraction weight (%) × DON concentration (mg/kg).

^C^ Calculated by total fraction recovery (mg/kg)/concentration at wheat kernel (mg/kg).

Several researchers reported that milling effectively reduces mycotoxin concentrations in wheat flours by 30% to 50% compared with that in cleaned grains[51]. Thus, removal of the wheat bran layer can decrease toxin levels[52–54]. Although milling cannot be solely used as an effective treatment to reduce DON from wheat grains, this technique can effectively decrease DON levels in the patent flour. However, the extent at which *Fusarium* toxins in cereal grains are removed during processing may vary based on the milling method employed[49].

**Fig 1:**
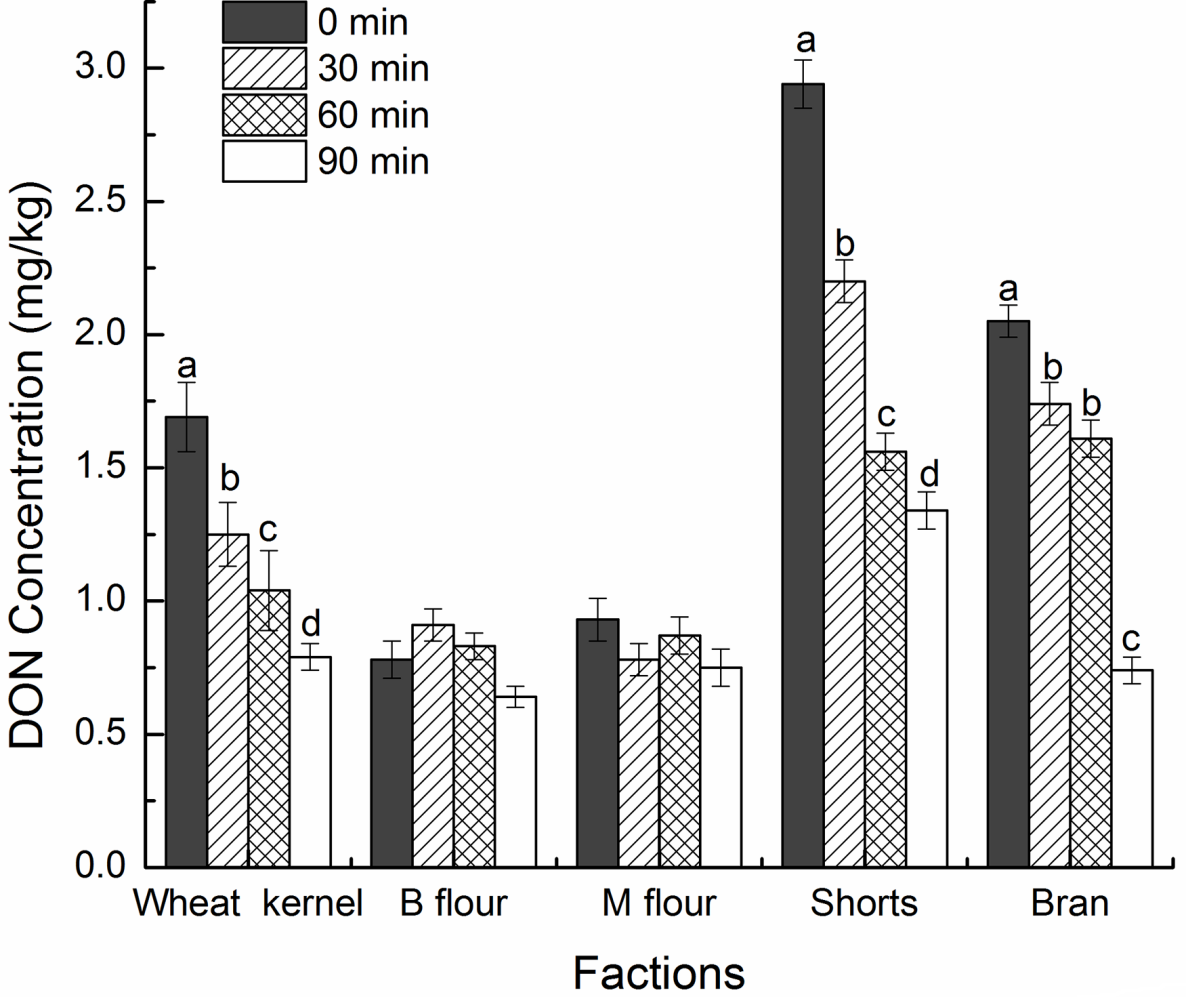
Distribution of DON in wheat kernel and milling fractions after ozone treatment. Values with different letters are significantly different (P < 0.05).

After 30, 60, or 90 min of exposure to 75 mg/L O_3_, the DON degradation rates in wheat kernels were 26.40%, 39.16%, and 53.48%, respectively (data not shown). The same effect was observed in bran and shorts, whereas degradation rate was not significant different among all flour fractions (Fig 1). Ozone gas significantly affected the external part of wheat grain compared with than in the endosperm when eliminating DON. These results are in agreement with the trends reported by Savi et al.[55]; in this study, ozone treatment significantly decreases DON level in the pericarp compared with that in the endosperm. In summary, DON levels decreased after milling because of exposure to ozone treatment.

According to Criegee[56], DON degradation occurs when ozone molecules undergo a 1–3 dipolar reaction with a double bond. This phenomenon leads to the formation of ozonides (1, 2, 4-trioxolanes) from alkenes and ozone with aldehyde or ketone oxides as decisive intermediates, all of which have finite lifetimes[57]. Previous studies demonstrated that ozone is an effective agent for detoxification of aflatoxins, as evidenced by the less toxicity of ozone-treated products than the untreated samples and the absence of mutagenic activities or deleterious effects. Reports also show that detoxification efficiency increases with increasing treatment time and ozone concentration[20,58]. However, few studies have focused on DON. The present study shows that ozonation can effectively detoxify DON in grains. DON is regulated in several countries because of its harmful effects on human and animal health. In China, the National Food Safety Standard of Maximum Levels of Mycotoxin in Foods (GB 2761–2012), which was issued in 2011, sets the maximum limit of 1 mg/kg for both wheat and flour. In the present study, both wheat kernel and flour samples treated with ozone can be used, thereby reducing the potential risk of illness compared with the untreated samples from the perspective of safety for consumption. In addition, ozone used before milling can enhance bran friability or the ability to separate bran from the starchy endosperm; ozone also significantly reduces (by 10%–20%) the required energy at the breaking stage without affecting grain hardness and flour yield[59].

### Protein and lipid analyses and alveographic measurements of wheat grains after ozone treatment

For quality assessment, parameters evaluated in this study included protein and fatty acid oxidation. Despite the limited lipid content (2.2%) of wheat grains, lipid oxidation may occur when ozone oxidizes the unsaturated lipids of the grain. The value of fatty acid was used as an indicator for evaluating the degree of oxidation by ozone. Apparently, the observed fatty acid value was not significantly different between treated and untreated groups (*P* > 0.05), indicating that lipid was not affected by ozone under the experimental conditions. We conclude that the degree of oxidation using this method was acceptable. This result is consistent with the findings of Naito[60], who reported that the oxidation of lipids in cereal grains, cereal grain powder, peas, and beans was rarely observed after 0.05 to 5 ppm ozone treatment for 1 h. Similarly, the effect on lipid oxidation was not apparent in peanuts, pistachio, and Brazil nuts after O_3_ application[58,61,62].

Wang et al.[63] found that the protein content of ozone-treated corn is lower than that of untreated corn; this finding indicated that protein could be destroyed by ozonation, thereby influencing the nutritional value of corn. Table 2 shows that no significant change (*P* > 0.05) occurred in protein content after the ozone treatment. These results are in accordance with the study by Mendez et al.[64], who reported that wheat treated with ozone showed no significant difference in protein content compared with that of untreated wheat. The difference between results of studies on wheat and corn may be attributed to the matrix effect. Corn was ground and thus porous, whereas the ozone cannot readily penetrate the whole wheat kernels.

**Table 2:** Changes in the physicochemical properties of ozone-treated wheat flour.

Exposure time (min)	Fatty acid value ^A^	Protein ^A^ (%)	W ^B^ (10E^-4^ J)	P/L ^B^
**0**	61.1 ± 1.3	14.5 ± 0.3	312^ab^	2.50^ab^
**30**	60.9 ± 0.4	15.6 ± 0.5	316^b^	2.69^b^
**60**	61.1 ± 0.9	16.6 ± 0.8	322^c^	3.26^c^
**90**	59.0 ± 0.8	14.8 ± 0.0	338^d^	3.48^d^

^A^ Values are presented as means ± standard deviations (*n* = 3) and are expressed on a dry weight basis.

^B^ Mean value of five experiments.

Values with different letters are significantly different (*P* < 0.05).

In the present study, all flours from ozone treated wheat exhibited W and P/L values higher than those in the control (W = 312.10E^−4^J, P/L = 2.50). This finding indicated that ozone treatment conferred flour with higher tenacity and lower extensibility (based on alveographic data) than native wheat. Gliadin is responsible for the viscous properties of dough, whereas glutenin contributes to dough strength. Disulfide bonds play a major role in determining dough properties. Exposure to oxidants can increase dough strength by oxidation of sulfhydryl groups to disulfide bonds[65]. We infer that disulfide bond formation is the main reaction underlying increased dough strength caused by ozone. Violleau et al.[32] also found that ozone treatment confers flour with higher force and tenacity and lower extensibility than those of the control. Ozone reacts with grain constituents (protein, lipid, and starch), depending on the concentration and duration of ozone exposure. Other variables, such as temperature, moisture, and grain characteristics, as well as the presence of other organic matter, such as insects and microorganisms on the grain surface, may also affect the diffusion of ozone into the grain.

### Color measurement of wheat flour after ozone treatment

The color of wheat flour is an important trait that strongly influences consumer acceptance of the flour itself and its end-products [66]. The color parameters (L*, a*, and b*) of the control and ozone-treated wheat flour samples are listed in Table 3. After ozone treatment, the L* value of wheat flour increased, whereas the a* and b* values significantly decreased; hence, ozone-treated wheat flour exhibited enhanced whiteness and reduced yellowness (*P* < 0.05). These results indicated that if ozone treatment was needed, such treatment would not decrease the acceptance of end users but can potentially improve the quality of flour. With regard to the color of wheat flour, ozone in gas or aqueous form can react with double bonds in carotenoid pigments, including β-carotene, xanthophyll, and flavones[16]. Previous studies also reported color changes in wheat flour. Chittrakorn[67] suggested that ozone could be used as a bleaching agent because flour treated with ozone gas is significantly less yellow and whiter than the control flour. Similar results were reported by Li et al.[66], who studied the effect of ozone treatment on the quality of wheat flour and shelf-life of fresh noodles. The current study demonstrated that the whiteness of flour and the noodle sheet increased after the ozone treatment.

**Table 3:** Color changes of ozone-treated wheat flour.

Exposure time (min)	L*	a*	b*
**0**	93.64 ± 0.12^a^	0.42 ± 0.02^a^	6.70 ± 0.02^a^
**30**	94.25 ± 0.02^b^	0.39 ± 0.00^b^	6.38 ± 0.05^bc^
**60**	94.23 ± 0.11^b^	0.36 ± 0.01^c^	6.27 ± 0.10^c^
**90**	94.18 ± 0.04^b^	0.38 ± 0.02^bc^	6.40 ± 0.09^b^

Values are presented as means ± standard deviations (*n* = 3). Values with different letters are significantly different (*P* < 0.05).

### Amino acid analysis

The nutritional quality of wheat is mainly related to the degree of balance of amino acids and essential amino acids. However, oxidation of amino acids by ozone can change the nutritional and metabolic value of grains, although few studies investigated this effect. Richard[68]_ENREF_1 reported that in aqueous solutions, amino acids that are readily oxidized by ozone include those containing the SH group, followed by tryptophan, tyrosine, and histidine. In the present study, the contents of essential amino acids (EAA) and total amino acids (TAA) slightly decreased; however, the ratio of EAA/TAA remained stable. In addition, no significant difference (*P* > 0.05) in tryptophan, tyrosine, or histidine was observed between the treated and untreated groups (Table 4). These results demonstrated that wheat treated under the experimental conditions were stable in terms of amino acid content. Similarly, Mendez et al.[64] indicated that treatment with 50 ppm ozone for 30 d did not change the amino acid content of hard and soft wheat, soybean, and maize. These data suggest that ozone did not adequately penetrate into the wheat kernel a less porous grain; thus, ozone did not reduce the nutritional value of the flour.

**Table 4:** Effect of ozone treatment on the amino acid content of wheat flour.

Amino acid (g/100 g)	Exposure time (min)			
	0	30	60	90
**Aspartic acid**	0.85 ± 0.13	0.66 ± 0.10	0.88 ± 0.15	0.91 ± 0.18
**Glutamic acid**	5.78 ± 1.10	5.70 ± 0.90	5.56 ± 1.30	5.60 ± 1.20
**Serine**	0.60 ± 0.04	0.53 ± 0.06	0.58 ± 0.05	0.55 ± 0.07
**Histidine**	0.40 ± 0.02	0.39 ± 0.03	0.38 ± 0.04	0.39 ± 0.03
**Glycine**	0.63 ± 0.05	0.59 ± 0.06	0.59 ± 0.05	0.59 ± 0.04
Threonine*	0.43 ± 0.02	0.40 ± 0.04	0.40 ± 0.04	0.41 ± 0.03
**Arginine**	0.84 ± 0.04	0.95 ± 0.06	0.87 ± 0.04	0.88 ± 0.03
**Alanine**	0.55 ± 0.03	0.52 ± 0.03	0.50 ± 0.05	0.49 ± 0.06
**Tyrosine**	0.34 ± 0.03	0.39 ± 0.02	0.36 ± 0.05	0.36 ± 0.04
**Cysteine**	0.09 ± 0.01	0.11 ± 0.03	0.11 ± 0.02	0.11 ± 0.03
Valine*	0.82 ± 0.05	0.78 ± 0.04	0.77 ± 0.03	0.77 ± 0.03
Methionine*	0.16 ± 0.03	0.21 ± 0.04	0.17 ± 0.03	0.19 ± 0.02
Phenylalanine*	0.82 ± 0.03	0.79 ± 0.05	0.77 ± 0.04	0.78 ± 0.03
Isoleucine*	0.70 ± 0.04	0.66 ± 0.05	0.64 ± 0.03	0.64 ± 0.04
Leucine*	1.16 ± 0.08	1.10 ± 0.07	1.09 ± 0.05	1.10 ± 0.06
Lysine*	0.35 ± 0.03	0.33 ± 0.02	0.33 ± 0.03	0.34 ± 0.02
**Proline**	2.01 ± 0.06	2.04 ± 0.05	1.85 ± 0.09	1.92 ± 0.09
Tryptophan*	0.13 ± 0.01	0.11 ± 0.03	0.16 ± 0.05	0.14 ± 0.04
**Essential amino acids**	4.57 ± 0.29	4.38 ± 0.34	4.34 ± 0.30	4.36 ± 0.27
**Total amino acids**	16.66± 1.80	16.26 ± 1.68	16.01 ± 2.14	16.17 ± 2.04
EAA/TAA**	0.27 ± 0.16	0.27 ± 0.20	0.27±0.14	0.27 ± 0.13

*Essential amino acid.

**Calculated by [Essential amino acids/Total amino acids].

Values are presented as means ± standard deviations (*n* = 3). All treatments were not statistically significant when compared with the control group (*P* > 0.05).

### Starch analysis

Carbonyl and carboxyl contents, which are essential components of starch, and the total starch content are listed in Table 5. The total contents of starch, carbonyl, and carboxyl of wheat starch did not significantly change with increasing ozone treatment duration (*P* > 0.05). Meanwhile, swelling power was not significantly different (*P* > 0.05) between the control and ozone-treated samples.

**Table 5:** Effect of ozone treatment on the starch composition of wheat flour.

Exposure time (min)	Starch (%)	Carbonyl content (%)	Carboxyl content (%)	Swelling power
0	60.6 ± 1.1	0.000	0.000	8.22 ± 0.11
30	60.0 ± 2.5	0.059 ± 0.012	0.014 ± 0.005	8.16 ± 0.08
60	57.0 ± 2.3	0.035 ± 0.014	0.010 ± 0.001	8.06 ± 0.06
90	65.0 ± 2.3	0.074 ± 0.036	0.011 ± 0.004	8.04 ± 0.13

Values are presented as means ± standard deviations (*n* = 3). All treatments were not statistically significant when compared with the control group (*P* > 0.05).

Sandhu et al.[31] reported that oxidizing agents can oxidize hydroxyl groups at C2, C3, and C6 positions on glucose molecules to the carbonyl and carboxyl groups. The introduction of carboxyl and carbonyl groups could increase swelling power and alter the pasting properties of starch[69]. The increase in the swelling power of ozone-oxidized starch is related to the introduction of hydrophilic carboxyl groups[70]. The negative charges of carboxyl groups repel each other, thereby enhancing the swelling of starch granules during heating in water[71]; this finding suggested that the carbonyl and carboxyl contents of waxy rice starch gradually increases with increasing ozone treatment duration. Sandhu et al.[31] also demonstrated an increase in carboxylic groups and swelling power in isolated wheat starch (50 g) upon exposure to ozone gas for 30 min. However, ozone treatment used in the present study did not cause any alteration in wheat starch, which could be due to the greater effect of ozone treatment on the external part of the wheat grain. Similarly, Savi et al.[55] stated that the carboxyl and carbonyl contents of wheat starch are only affected by ozone treatment after 180 min. Other variables, such as botanical origin of starch, type of oxidizing agent, and reaction conditions, may result in differences in starch carboxyl and carbonyl contents, as discussed by Sangseethong et al.[72].

## Conclusion

This study evaluated DON distribution in wheat kernel, effect of ozone treatment on DON degradation in wheat kernels, and changes in protein, lipid, dough quality, amino acids, color, and starch of wheat flour. A high toxin concentration was found in the outer part of the kernel, and the DON levels were higher inside than that outside the kernel. At a processing time of 90 min and ozone concentration of 75 mg/L, the DON degradation rate reached 53.48%. Ozone induced a more significant effect on the external part than that on the endosperm of wheat grains. No significant changes were observed in terms of protein content, fatty acid value, amino acid content, starch content, carbonyl and carboxyl contents, and swelling power of samples after ozone treatment. The tenacity and whiteness of the starch were higher, and its extensibility and yellowness were lower than those in the control samples.

Overall, this study reveals that ozone exhibits a remarkable potential in reducing fungal growth and removing various mycotoxins in wheat grains but minimally affects the quality of wheat naturally contaminated with DON. This study also provides information for potential users of ozone-treated grain. Future investigations are needed to determine the economic benefits of implementing ozone technology as a part of an integrated management program to remove various mycotoxins in wheat grains and study the degradation products of DON and their structures. Moreover, the mechanism for detoxifying DON, particularly in the wheat sample, can be elucidated. From the perspective of safety for consumption, the toxicity of ozone-treated wheat will be investigated through sub-chronic toxicity experiments.

## References

[1] Abbas HK MC, Pawlosky RJ, Pusch DJ. (1985) Effect of cleaning, milling, and baking on deoxynivalenol in wheat. Applied and Environmental Microbiology 50: 482–486. 4051489 10.1128/aem.50.2.482-486.1985 PMC238646

[2] Tanaka T, Hasegawa A, Yamamoto S, Lee US, Sugiura Y, Ueno Y (1988) Worldwide contamination of cereals by the Fusarium mycotoxins nivalenol, deoxynivalenol, and zearalenone. 1. Survey of 19 countries. Journal of Agricultural and Food Chemistry 36: 979–983.

[3] Parry DW, Jenkinson P, Mcleod L (1995) Fusariumear blight (scab) in small grain cereals, a review. Plant Pathology 44: 207–238.

[4] Zheng Y, Hossen SM, Sago Y, Yoshida M, Nakagawa H, Nagashima H, et al (2014) Effect of milling on the content of deoxynivalenol, nivalenol, and zearalenone in Japanese wheat. Food Control 40: 193–197.

[5] Rotter BA (1996) Invited review: Toxicology of deoxynivalenol (vomitoxin). Journal of Toxicology and Environmental Health Part A 48: 1–34. 10.1080/009841096161447 8637056

[6] Wijnands LM, Van Leusden FM. RIVM report 257852 004. (2000) An overview of adverse health effects caused by mycotoxins and biossays for their detection.

[7] Pestka JJ (2007) Deoxynivalenol: toxicity, mechanisms and animal health risks. Animal feed science and technology 137: 283–298.

[8] IARC (1993) Some naturally occurring substances: Food items and constituents, heterocyclic aromatic amines and mycotoxins Lyon, France: World Health Organization pp. 397–444.

[9] Huwig A, Freimund S, Kappeli O, Dutler H (2001) Mycotoxin detoxication of animal feed by different adsorbents. Toxicology Letters 122: 179–188. 11439224 10.1016/s0378-4274(01)00360-5

[10] Bata A, Lasztity R (1999) Detoxification of mycotoxin-contaminated food and feed by microorganisms. Trends in Food Science & Technology 10: 223–228.

[11] Young JC, Subryan LM, Potts D, McLaren ME, Gobran FH (1986) Reduction in levels of deoxynivalenol in contaminated wheat by chemical and physical treatment. Journal of Agricultural and Food Chemistry 34: 461–465.

[12] Blandino M, Minelli L, Reyneri A (2006) Strategies for the chemical control of Fusarium head blight: Effect on yield, alveographic parameters and deoxynivalenol contamination in winter wheat grain. European Journal of Agronomy 25: 193–201.

[13] Khadre MA, Yousef AE, Kim JG (2001) Microbiological aspects of ozone applications in food: a review. Journal of Food Science 66: 1242–1252.

[14] Register F (2001) Secondary direct food additives permitted in food for human consumption. Federal Register 66: 33829–33830.

[15] Nations. FaAOotU (2004) Food outlook. Report no.1. Available at http://www.fao.org/docrep/006/J2084e/j2084e00.htm.

[16] Sandhu HPS, Manthey FA, Simsek S (2011) Quality of bread made from ozonated wheat (Triticum aestivum L.) flour. Journal of the Science of Food and Agriculture 91: 1576–1584. 10.1002/jsfa.4350 21445841

[17] de Alencar ER, Faroni LRD, Soares NDF, da Silva WA, Carvalho MCD (2012) Efficacy of ozone as a fungicidal and detoxifying agent of aflatoxins in peanuts. Journal of the Science of Food and Agriculture 92: 899–905. 10.1002/jsfa.4668 22095762

[18] McDonough MX, Campabadal CA, Mason LJ, Maier DE, Denvir A, Woloshuk C (2011) Ozone application in a modified screw conveyor to treat grain for insect pests, fungal contaminants, and mycotoxins. Journal of Stored Products Research 47: 249–254.

[19] Tiwari B, Brennan CS, Curran T, Gallagher E, Cullen P, O'Donnell C (2010) Application of ozone in grain processing. Journal of Cereal Science 51: 248–255.

[20] Inan F, Pala M, Doymaz I (2007) Use of ozone in detoxification of aflatoxin B-1 in red pepper. Journal of Stored Products Research 43: 425–429.

[21] Ibanoglu S (2001) Influence of tempering with ozonated water on the selected properties of wheat flour. Journal of Food Engineering 48: 345–350.

[22] Zorlugenc B, Zorlugenc FK, Oztekin S, Evliya IB (2008) The influence of gaseous ozone and ozonated water on microbial flora and degradation of aflatoxin B(1) in dried figs. Food and Chemical Toxicology 46: 3593–3597. 10.1016/j.fct.2008.09.003 18824207

[23] Dwarakan Ct, Rayner ET, Mann GE, Dollear FG (1968) Reduction of Aflatoxin Levels in Cottonseed and Peanut Meals by Ozonization. Journal of the American Oil Chemists Society 45: 93–95. 5694281 10.1007/BF02890715

[24] Proctor AD, Ahmedna M, Kumar JV, Goktepe I (2004) Degradation of aflatoxins in peanut kernels/flour by gaseous ozonation and mild heat treatment. Food Additives and Contaminants 21: 786–793. 15370830 10.1080/02652030410001713898

[25] Caulfield MJ, Burleson GR, Pollard M (1979) Ozonation of Mutagenic and Carcinogenic Alkylating-Agents, Pesticides, Aflatoxin-B1, and Benzidine in Water. Cancer Research 39: 2155–2159. 445412

[26] Diao EJ, Shan CP, Hou HX, Wang SS, Li MH, Dong HZ (2012) Structures of the Ozonolysis Products and Ozonolysis Pathway of Aflatoxin B-1 in Acetonitrile Solution. Journal of Agricultural and Food Chemistry 60: 9364–9370. 10.1021/jf302528e 22920447

[27] Luo XH, Wang R, Wang L, Wang Y, Chen ZX (2013) Structure elucidation and toxicity analyses of the degradation products of aflatoxin B-1 by aqueous ozone. Food Control 31: 331–336.

[28] Luo XH, Wang R, Wang L, Li YF, Bian YY, Chen ZX (2014) Effect of ozone treatment on aflatoxin B-1 and safety evaluation of ozonized corn. Food Control 37: 171–176.

[29] Young JC, Zhu HH, Zhou T (2006) Degradation of trichothecene mycotoxins by aqueous ozone. Food and Chemical Toxicology 44: 417–424. 16185803 10.1016/j.fct.2005.08.015

[30] Li MM, Guan EQ, Bian K (2014) Effect of ozone treatment on deoxynivalenol and quality evaluation of ozonised wheat. Food Additives & Contaminants: Part A: 544–553. 10.1080/19440049.2014.976596 25325346

[31] Sandhu HPS, Manthey FA, Simsek S (2012) Ozone gas affects physical and chemical properties of wheat (Triticum aestivum L.) starch. Carbohydrate Polymers 87: 1261–1268.

[32] Violleau F, Pernot AG, Surel O (2012) Effect of Oxygreen (R) wheat ozonation process on bread dough quality and protein solubility. Journal of Cereal Science 55: 392–396.

[33] AACC (2000) Approved methods of the American Association of Cereal Chemists (10th ed). American Association of Cereal Chemists. St. Paul, MN: AACC International.: International Agency for Research on Cancer.

[34] GB/T (2009) Determination of Deoxynivalenol In Food-High Performance Liquid Chromatographic Method With Immunoaffinity Column Clean-up.

[35] ISO 5530–4 (1991). Wheat flour—Physical characteristics of doughs—Part 4: Determination of rheological properties using an alveograph.

[36] Indrani D, Manohar RS, Rajiv J, Rao GV (2007) Alveograph as a tool to assess the quality characteristics of wheat flour for parotta making. Journal of Food Engineering 78: 1202–1206.

[37] Rose DJ, Ogden LV, Dunn ML, Pike OA (2008) Enhanced lipid stability in whole wheat flour by lipase inactivation and antioxidant retention. Cereal chemistry 85: 218–223.

[38] Zhong Y, He C, Chen C (2008) Determination of amino acids in foods by high performance liquid chromatography. China Tropical Medicine: 132–134.

[39] Rupollo G, Vanier NL, da Rosa Zavareze E, de Oliveira M, Pereira JM, Paraginski RT, et al (2011) Pasting, morphological, thermal and crystallinity properties of starch isolated from beans stored under different atmospheric conditions. Carbohydrate Polymers 86: 1403–1409.

[40] Vanier NL, da Rosa Zavareze E, Pinto VZ, Klein B, Botelho FT, Dias ARG, et al (2012) Physicochemical, crystallinity, pasting and morphological properties of bean starch oxidised by different concentrations of sodium hypochlorite. Food chemistry 131: 1255–1262.

[41] Chattopadhyay S, Singhal RS, Kulkarni PR (1997) Optimisation of conditions of synthesis of oxidised starch from corn and amaranth for use in film-forming applications. Carbohydrate Polymers 34: 203–212.

[42] Smith RJ (1967) Production and use of hypochlorite oxidized starches. Starch chemistry and technology New York: Academic Press pp. 620–625.

[43] McCormick KPJ, Hong S. (1991) A swelling power test for selecting potential noodle quality wheats. Crop and Pasture Science 42: 317–323.

[44] Young JCFR, Hayhoe JH, Scott PM, Dexter JE (1984) Effect of milling and baking on deoxynivalenol (vomitoxin) content of eastern Canadian wheats. Journal of Agricultural and Food Chemistry 32: 659–664.

[45] Lancova K, Hajslova J, Kostelanska M, Kohoutkova J, Nedelnik J, Moravcova H, et al (2008) Fate of trichothecene mycotoxins during the processing: Milling and baking. Food Additives & Contaminants Part A: Chemistry, Analysis, Control, Exposure & Risk Assessment 25: 650–659. 10.1080/02652030701660536 18473219

[46] Katta SK, Cagampang AE, Jackson LS, Bullerman LB (1997) Distribution of Fusarium Molds and Fumonisins in Dry-Milled Corn Fractions 1. Cereal Chemistry 74: 858–863.

[47] Park DL (2002) Effect of processing on aflatoxin Mycotoxins and Food Safety: Springer pp. 173–179.

[48] Brera C, Debegnach F, Grossi S, Miraglia M (2004) Effect of industrial processing on the distribution of fumonisin B1 in dry milling corn fractions. Journal of Food Protection® 67: 1261–1266. 15222562 10.4315/0362-028x-67.6.1261

[49] Zhang H, Wang B (2014) Fate of deoxynivalenol and deoxynivalenol-3-glucoside during wheat milling and Chinese steamed bread processing. Food Control 44: 86–91.

[50] Jackson LS, Voss KA, Ryu D (2012) Effects of different extrusion conditions on the chemical and toxicological fate of fumonisin B1 in maize a short review. World Mycotoxin Journal 5: 251–260.

[51] Dexter JE, Clear RM, Preston KR (1996) Fusarium head blight: effect on the milling and baking of some Canadian wheats. Cereal Chemistry 73: 695–701.

[52] Cabañas R, Bragulat MR, Abarca ML, Castellá G, Cabañes FJ (2008) Occurrence of Penicillium verrucosum in retail wheat flours from the Spanish market. Food microbiology 25: 642–647. 10.1016/j.fm.2008.04.003 18541161

[53] Lee US, Jang HS, Tanaka T, Oh YJ, Cho CM, Ueno Y (1987) Effect of milling on decontamination of Fusarium mycotoxins nivalenol, deoxynivalenol and zearalenone in Korean wheat. Journal of Agricultural and Food Chemistry 35: 126–129

[54] Visconti A, Haidukowski EM, Pascale M, Silvestri M (2004) Reduction of deoxynivalenol during durum wheat processing and spaghetti cooking. Toxicology letters 153: 181–189. 15342095 10.1016/j.toxlet.2004.04.032

[55] Savi GD, Piacentini KC, Bittencourt KO, Scussel VM (2014) Ozone treatment efficiency on Fusarium graminearum and deoxynivalenol degradation and its effects on whole wheat grains (Triticum aestivum L.) quality and germination. Journal of Stored Products Research 59: 245–253.

[56] Criegee R (1975) Mechanism of ozonolysis. Angewandte Chemie International Edition in English 14: 745–752.

[57] Cullen PJ, Tiwari BK, O'Donnell CP, Muthukumarappan K (2009) Modelling approaches to ozone processing of liquid foods. Trends in Food Science & Technology 20: 125–136.

[58] Akbas MY, Ozdemir M (2006) Effect of different ozone treatments on aflatoxin degradation and physicochemical properties of pistachios. Journal of the Science of Food and Agriculture 86: 2099–2104.

[59] Desvignes C, Chaurand M, Dubois M, Sadoudi A, Abecassis J, Lullien-Pellerin V (2008) Changes in common wheat grain milling behavior and tissue mechanical properties following ozone treatment. Journal of Cereal Science 47: 245–251.

[60] Naito S (1989) The influence of ozone treatment on lipids contained in cereal grains, cereal grain powders, peas, beans and pulse products. Nippon Shokuhin Kogyo Gakkaishi 36: 878–883.

[61] Scussel VM, Giordano BN, Simao V, Manfio D, Galvao S, Rodrigues MNF (2011) Effect of oxygen-reducing atmospheres on the safety of packaged shelled Brazil nuts during storage. International journal of analytical chemistry 1687–8760. 10.1155/2011/813591 PMC3132530 21760791

[62] Chen R, Ma F, Li PW, Zhang W, Ding XX, Zhang Q, et al (2014) Effect of ozone on aflatoxins detoxification and nutritional quality of peanuts. Food Chemistry 146: 284–288. 10.1016/j.foodchem.2013.09.059 24176344

[63] Wang Y, King JM, Xu ZM, Losso J, Prudente A (2008) Lutein from ozone-treated corn retains antimutagenic properties. Journal of Agricultural and Food Chemistry 56: 7942–7949. 10.1021/jf801562v 18681445

[64] Mendez F, Maier DE, Mason LJ, Woloshuk CP (2003) Penetration of ozone into columns of stored grains and effects on chemical composition and processing performance. Journal of Stored Products Research 39: 33–44.

[65] Yeh A-I SS Y (1999) Effects of Oxido-reductants on rheological properties of wheat flour dough and comparison with some characteristics of extruded noodles. Cereal Chemistry 76: 614–620.

[66] Li M, Peng J, Zhu KX, Guo XN, Zhang M, Peng W, et al (2013) Delineating the microbial and physical-chemical changes during storage of ozone treated wheat flour. Innovative Food Science & Emerging Technologies 20: 223–229.

[67] Chittrakorn S (2008) Use of ozone as an alternative to chlorine for treatment of soft wheat flours: ProQuest.

[68] Richard Y, Brener L (1984) Removal of ammonia and other nitrogen derivatives from drinking water with ozone. Handbook of Ozone Technology and Applications.

[69] Chan HT, Bhat R, Karim AA (2009) Physicochemical and functional properties of ozone-oxidized starch. Journal of agricultural and food chemistry 57: 5965–5970. 10.1021/jf9008789 19489606

[70] Kuakpetoon D, Wang YJ (2001) Characterization of different starches oxidized by hypochlorite. Starch‐Stärke 53: 211–218.

[71] Ding W, Wang Y, Zhang W, Shi Y, Wang D (2015) Effect of ozone treatment on physicochemical properties of waxy rice flour and waxy rice starch. International Journal of Food Science & Technology 50: 744–749.

[72] Sangseethong K, Termvejsayanon N, Sriroth K (2010) Characterization of physicochemical properties of hypochlorite-and peroxide-oxidized cassava starches. Carbohydrate Polymers 82: 446–453.

